# Scoring consistency of standard patients and examiners in the developed dental objective structured clinical examination system

**DOI:** 10.1186/s12909-023-04087-6

**Published:** 2023-02-17

**Authors:** Feng Zhu, Li Wu, Xiuxiu Shao, Lijuan Huang, Xiangfeng Meng, Rongrong Nie

**Affiliations:** 1 Department of oral and maxillofacial surgery, Nanjing Stomatological Hospital, Medical School of Nanjing University, Nanjing, China; 2 Department of Education, Nanjing Stomatological Hospital, Medical School of Nanjing University, Nanjing, China; 3 Department of Prosthodontics, Nanjing Stomatological Hospital, Medical School of Nanjing University, Nanjing, China

**Keywords:** Consistency, Dental, Objective structured clinical examination, Residents, Standard patient

## Abstract

**Objective:**

To investigate the role of standard patients (SPs) and examiners as assessors for scoring in the dental objective structured clinical examination (OSCE) system and to evaluate the scoring differences between them.

**Methods:**

We developed the doctor-patient communication and clinical examination station in the OSCE system. The examination time of this station was 10 min, and the examination institution wrote the script and recruited SPs. A total of 146 examinees who received standardized resident training at the Nanjing Stomatological Hospital, Medical School of Nanjing University between 2018 and 2021 were assessed. They were scored by SPs and examiners according to the same scoring rubrics. Subsequently, the SPSS software was used to analyze the examination results of different assessors and evaluate the consistency.

**Results:**

The average score of all examinees provided by SPs and examiners was 90.45 ± 3.52 and 91.53 ± 4.13, respectively. The consistency analysis showed that the intraclass correlation coefficient was 0.718, which was indicative of medium consistency.

**Conclusion:**

Our findings showed that SPs could be used directly as assessors, as they could provide a simulated and realistic clinical setting and create favorable conditions for comprehensive competence training and improvement for medical students.

## Introduction

Objective structured clinical examination (OSCE), a multi-station clinical skill examination, is an approach used for assessing the clinical competence of medical students [1, 2]. This type of assessment is mainly based on a series of pre-designed simulated clinical settings, which are used to evaluate the clinical competence of medical students. The examinees are required to complete the tasks designed at each station and are assessed at multiple stations simulating clinical settings. Since the 1990s, OSCE has been included in the curricula of several dental schools worldwide to assess the competence of dental school students in various parameters, including communication, patient education, clinical skills, and critical thinking [3, 4].

A doctor-patient communication and clinical examination station are important in the OSCE used to conduct standardized and systematic training for healthy individuals to help them pose as standard patients (SPs). Furthermore, SPs can accurately present actual clinical problems and imitate the symptoms of the corresponding cases, including body movements, pain degree, facial expressions, self-report of symptoms in the medical history, and so on. The examinees establish a correct diagnosis based on SP’s medical history, conditions, and symptoms. During the examination, SP principally takes the role of a patient who “has medical history and undergoes physical examinations.” Also, a well-trained SP can act as an examiner to assess examinees’ performance and even guide them. Thus, the recruitment of SP allows dental school students to practice communication skills and obtain patients’ feedback [5].

Compared with other assessment methods, the “objectification” and “structuration” of OSCE can well simulate real clinical settings and achieve consistency in assessment contents. However, OSCE requires numerous examiners and other examination personnel to develop detailed pre-exam plans during the actual implementation. The limitations related to examination time, space, and the lack of relevant examination staff are the common factors affecting the promotion and implementation of OSCE [6, 7]. Whether SP can take the role of the examiner of doctor-patient communication and clinical examination station to directly score candidates and give feedback in OSCE is worth investigating. Despite some initial concern that SPs are not dentists and their judgment might lack reliability and validity, numerous studies have demonstrated that SPs scoring performance was as reliable as that of professional examiners, particularly for characteristics related to communication and professionalism, provided they are adequately trained [8–10].

The present study aimed to construct a suitable doctor-patient communication and clinical examination station, improving the rating scale and pre-exam training. The SP undertook the role of an examiner, providing feedback and guidance to the examinees, thus achieving an environment similar to clinical practice and reducing the involvement of a large number of examiners in the OSCE . In the present study, the scoring differences between SPs and stomatological examiners were assessed based on a 4-year resident midterm evaluation.

## Methods

### Participants and methods

The present study was approved by the Institutional Review Board of Nanjing Stomatological Hospital, Medical School of Nanjing University (No. NJSH-2022NL-074). The OSCE system was used for the midterm assessment of 146 residents in the aforementioned hospital between 2018 and 2021. These residents were in the second year of standardized resident training and completed basic theoretical courses and 1 year of clinical practice during the examination. An SP and an examiner scored all examinees according to the same scoring rubrics, who wrote brief feedback on the rating scale for each examinee. The study was conducted in strict accordance with the requirements of the Declaration of Helsinki. The participation was voluntary, without any compensation or incentive. Both confidentiality and anonymity were guaranteed for all participants. Participating residents filled out an informed consent form.

### Construction of the SP station

The OSCE assessment system has been used for dental teaching evaluation since 2016. The OSCE had eight stations; the third was the doctor-patient communication and clinical examination station. SP was required to assess students’ doctor-patient communication and clinical examination competencies and whether the students could perform standardized receptions. The SPs were also required to comprehensively and accurately record the medical history based on chief complaints, make a possible diagnosis, and determine further examination and treatment plans in combination with the clinical examination results. Meanwhile, the students’ communication skills, such as appearance, attitude, and language expression during clinical reception, as well as inquiry of patients, diagnosis and treatment decisions, disease prognosis, diagnosis and treatment costs, and similar, were assessed (Table 1). In this station, the examination time was 10 min, and the score was calculated using the centesimal system, accounting for 8% of the total score [11].

### Recruitment of SPs

Four scripts were written based on the actual dental conditions i.e., acute pulpitis, pericoronitis of wisdom teeth, gingivitis, and tooth defects. Oral examinations were performed on the voluntary participants at this hospital every year 1 week before the examination. The volunteers, usually college students from other schools, were screened out by oral detection following the script requirements, and SPs were preferentially selected according to their own wishes. Two SPs were recruited for each script, totaling eight SPs. The rotation was made every 2 h during the examination.

### Pre-exam training

**Fig. 1:**
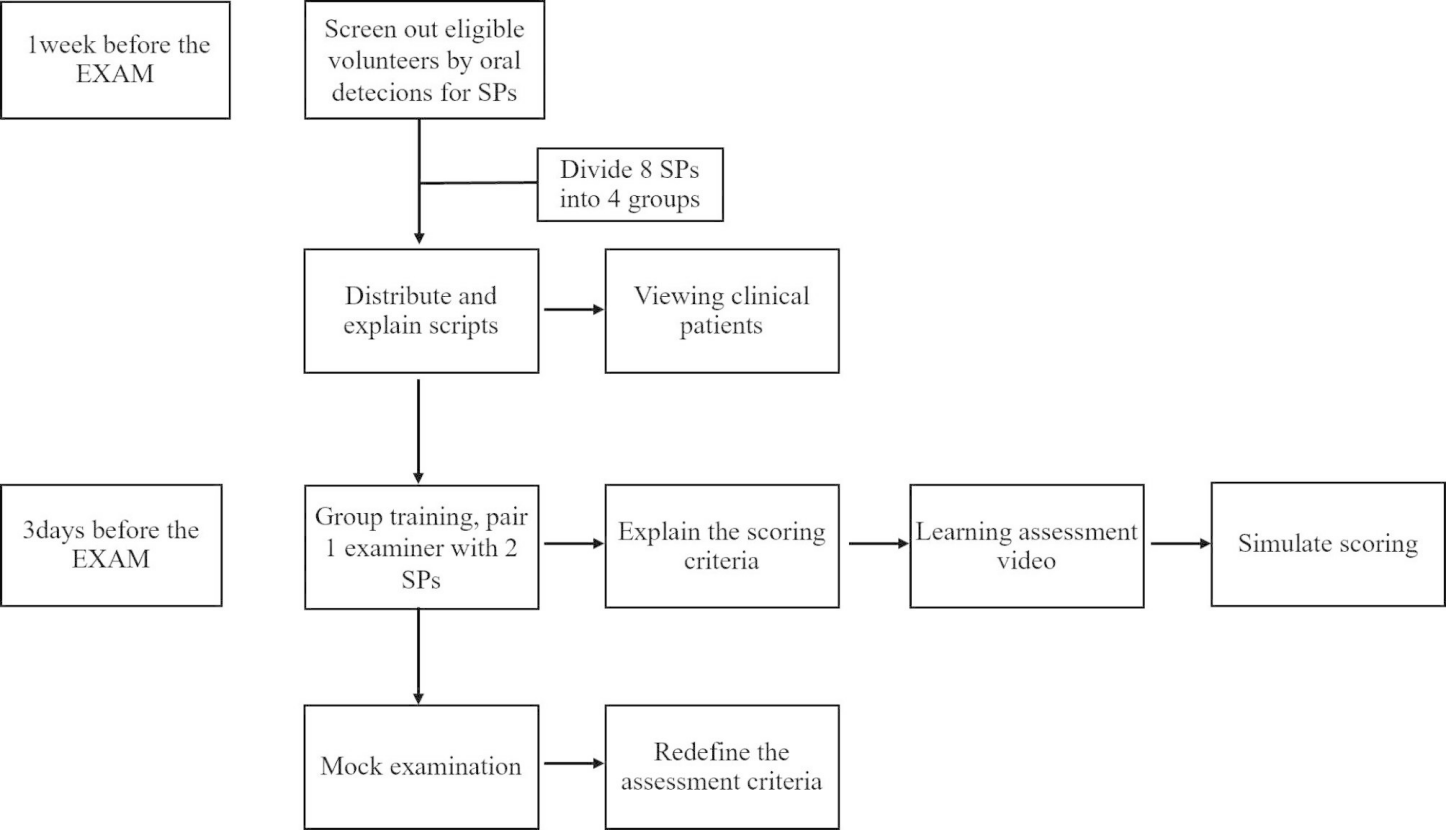
Training process before exam for SPs and examiners

Teachers with experience in SP training distributed and explained their scripts and watched real clinical patient videos after SP recruitment. SPs and station examiners were recruited 3 days before the examination. Four examiners were teachers with more than 3 years of teaching experience. Each examiner corresponded to two SPs of the same script, which were assigned to four groups. In addition, the chief examiner in charge of the SP station explained the scoring rubrics to SPs and examiners according to the rating scale, reviewed the assessment videos of the previous year, and conducted simulated scoring (Fig. 1). At the same time, eight simulated examinees performed field training, while SPs performed their task and scored with examiners. Finally, the passing criteria were explained again.

### Statistical analysis

All measurement data were expressed using $\overline{\overline x} \pm {\rm{SD}}$ said. Since the scores were not normally distributed, the data were compared by Wilcoxon signed-rank test. Intraclass correlation coefficient (ICC) was used to analyze 2-way random effects and the consistency of scoring results among different raters. ICC ≤ 0.4 was considered poor consistency, 0.4 < ICC < 0.75 was considered moderate consistency, and ICC ≥ 0.75 was considered good consistency. A p value < 0.05 was taken as the threshold for statistical difference. SPSS 26.0 software was used for all statistical analyses.

**Table 1:** Rating scale of the doctor-patient communication and clinical examination station

Item		Requirements	Score	Points
Doctor-patient communication (50)	Professional behavior	Neatly dressed in a standardized manner and meeting professional requirements	10	
	Personal emotion	Display various behaviors when interacting with patients, including gestures, facial expressions, or confidence during a conversation	10	
	Language communication	Able to use simple language to explain patient’s problems and ask about relevant medical history for chief complaints during communication	10	
	Relationship building	Build a harmonious relationship with the patient and express comfort and care about the patient’s disease experience so that the patient is willing to communicate	10	
	Patient management	Provide hygiene guidance to patients and propose further treatment plans and recommendations	10	
Clinical examination (50)	Preparation before examination	Promote proper hand washing, wearing gloves, and adjusting the chair position to keep the patient in a comfortable position	10	
	Communication during the examination	Inform the patient before the examination and pay attention to the patient’s feelings during the examination	10	
	Examination methods	Select appropriate examination instruments and perform examinations in a correct manner	10	
	Consciousness of patient-friendly and dedication	Be careful not to cause pain and discomfort to the patient during the examination	10	
	End of examination	Take off gloves and wash hands at the end of the examination; adjust the chair to make it easier for the patient to get up and leave the premises	10	

## Results

The score results of different raters for the 10 items did not conform to normal distribution. Wilcoxon signed-rank test showed that the score results of different raters for items “Patient management”, “Preparation before examination”, “End of examination”, and total score significantly differed (Table 2). Table 3 shows the consistency of scores of 10 items and the total scores by SPs and examiners. Although the ICC of the total scores was 0.718, indicating moderate consistency, we noticed that the ICC of 5 items was close to 0.4, which indicated poor consistency, especially for some items that relied on subjective feelings.

**Table 2:** Scores of SPs and examiners

Item	Score			Z	*P*
	SP	Examiner			
Professional behavior	9.70 ± 0.647		9.66 ± 0.698	-0.712	0.477
Personal emotion	9.43 ± 0.674		9.48 ± 0.763	-1.013	0.311
Language communication	8.31 ± 1.067		8.48 ± 1.463	-1.734	0.083
Relationship building	9.58 ± 0.820		9.65 ± 0.802	-0.858	0.391
Patient management	7.79 ± 1.361		7.99 ± 1.407	-3.351	0.001^*^
Preparation before examination	9.07 ± 0.802		9.27 ± 0.891	-3.023	0.003^*^
Communication during the examination	9.08 ± 0.933		9.20 ± 0.937	-1.922	0.055
Examination methods	9.34 ± 0.934		9.36 ± 0.967	-0.284	0.776
Consciousness of patient-friendly and dedication	9.10 ± 0.764		9.24 ± 0.833	-1.901	0.057
End of examination	9.06 ± 0.726		9.20 ± 0.711	-2.107	0.035^*^
Total	90.45 ± 3.516		91.53 ± 4.129	-4.276	0.001^*^

^*^Represents *P <* 0.05

**Table 3:** Consistency analysis for 10 items and total scores of SPs and examiners

Item	Intraclass Correlation	95% Confidence interval		*Value*	*P*
		lower bound	upper bound		
Professional behavior	0.674	0.575	0.754	5.131	< 0.001
Personal emotion	0.464	0.326	0.582	2.725	< 0.001
Language communication	0.429	0.287	0.552	2.513	< 0.001
Relationship building	0.469	0.332	0.586	2.764	< 0.001
Patient management	0.858	0.8	0.899	14.045	< 0.001
Preparation before examination	0.545	0.415	0.652	3.538	< 0.001
Communication during the examination	0.665	0.564	0.747	5.049	< 0.001
Examination methods	0.586	0.468	0.683	3.811	< 0.001
The consciousness of patient-friendly and dedication	0.391	0.246	0.519	2.309	< 0.001
End of examination	0.411	0.268	0.537	2.431	< 0.001
Total	0.718	0.586	0.805	6.855	< 0.001

## Discussion

The widespread application of SPs in dental education reflects continuous efforts to improve the humanistic quality of health care. Trained SPs can teach students how to communicate with patients and deal with unexpected problems, provide humanistic health care, and improve their abilities to identify, analyze, and deal with problems. The clinical thinking abilities of dental students can be improved through inquiry and physical examinations of SP, and by communicating with SPs, understanding patients’ experience of illness, and providing health education to patients [12]. On the other hand, SPs can assess students, record and identify their shortcomings, and provide them with a realistic and comprehensive clinical process and real feelings through personal experience combined with the actual situation and scoring rubrics. The involvement of SPs under the framework of OSCE further enhances the assessment, making it more rational and objective. Furthermore, each SP is trained for one aspect so that each SP faces the same problems, making the evaluation impartial and accurate, thus avoiding the previous biases caused by recording medical histories and signs of different patients by different students.

OSCE is designed to standardize the examination and reduce variables that may affect performance assessment. Thus, the examination results of examinees are mainly affected by their competencies for a well-designed OSCE, ensuring minimal interference from other variances. The consistency of SP performance for each examinee is crucial for the SP station. Poorly standardized SPs can perform differently for different examinees, thus reducing the reliability of the examination [13]. Therefore, the training of examiners and SPs is important in the quality assurance and standardization process before the examination [14]. Due to the commonness of oral diseases, we screened out the oral diseases of SPs before the exam and treated them actively after the exam for humanitarian reasons. Therefore, we could not train a group of SPs for long-term assessment. The assessment content about the examination station and the training before the examination was crucial as a new group of SPs had to be trained for each examination.

First, in terms of the content and purpose of the examination, we aimed to reduce the number of examiners, score candidates, and provide feedback through SPs, thus assessing students’ clinical communication skills. The examination items mainly focused on the interaction between candidates and patients rather than on the professional operation. Relatively basic consciousness of patient-friendly and nosocomial infection prevention and control could increase the sense of trust between doctors and patients. Researchers also argued that SP, as a direct participant in this site, could interact with candidates, as they could determine the differences between candidates in a more detailed manner to other professional operational assessment [15, 16]. Transferring some marking items from examiners to SPs and combining the SP and examiner scores can improve the reliability of the entire OSCE [17].

In the present study, SPs were trained three times before the examination, and SPs and matched examiners were trained two times. Besides explaining scripts and scoring rubrics, the training also analyzed previous examination videos and organized the training of simulated examinees. A checklist with detailed contents, i.e., the assessment points of examinees in communication, was designed to increase the consistency of the assessment. These measures could reduce the differences in examiners and improve behavioral consistency through careful pre-exam design and training, regardless of examiners, thereby increasing the reliability of the examination [18]. According to the results, no significant difference was found between the scores of SP and examiner in most items. In the four years from 2018 to 2021, there was a high degree of consistency between the two scores, with ICC reaching 0.718.

The selection of SP as the station examiner can reduce the number of examiners at OSCE. More importantly, real feedback can be obtained from patients. A previous study showed that medical students found direct feedback from patients as the most valuable part of SP training assessment, as was also confirmed by the following statement: “This is a very rare opportunity because we rarely obtain this kind of feedback from real patients and their families” [19]. The assessment also showed that the SPs were more likely to detect some details that might be neglected by the examiners, such as “he (she) does not look at me during the communication,” “I often get interrupted while talking,” “he (she) is unable to empathize with my pain,” “the movement during the examination is harsh,” “the explanation of the conditions is too specialized,” and so forth. As professionals, the examiners might be familiar with disease settings, underestimating the importance of comfort and professional explanation. At the same time, as a bystander, the examiners could not share the patients’ experiences gained during the interaction with the examinees. This could explain the poor consistency of scores between SPs and examiners in many items. As an examination station designed for assessing the relationship between doctors and patients, we believe that this station should focus on patients’ most direct feelings and experiences rather than professional knowledge.

The rapid development of modern digital virtual technology has made it possible to use virtual patients in medical education to simulate some clinical scenes that SP cannot replicate and also design more disease situations, such as maxillofacial fractures and tumors. Digital education can be flexibly used for unlimited durations compared with traditional SP role-playing. It can also save time, workforce, and space resources. Also, following the outbreak of Corona Virus Disease-19(COVID-19), the assessment is more likely to be conducted through remote video or picture recording. However, previous studies showed that encounters with SP may cause anxiety among examinees, which was a stress-related response [20, 21]. In their study, Luctkar-Flude showed that when SPs replaced examiners, the candidates felt more pressure and anxiety due to their communication with “real” patients [22], which affected their performance. Therefore, the use of SP is closer to the real clinical environment as it emotionally prepares candidates to deal with similar clinical events in real life, which cannot be replaced by digital technology. However, we also reflected on the current SP recruitment and training process, finding that oral conditions of SP patients tend to change, so SP training cannot be stable for a long time, and the need for training courses cannot be guaranteed. The diversity of diseases is also greatly limited. Therefore, we are currently trying to continue using SP for doctor-patient communication while using virtual scenarios for clinical examination. In this way, we can overcome the limitation of the actual dental condition of the SP, cultivate long-term cooperation with SP, and further reduce the impact of inadequate training and frequent replacement of SP on the accuracy of the examination. It is hoped that the cooperation between real SP and virtual technology can achieve better results.

The limitations of the present study included a small sample size of students and examiners and a shorter observation time; hence, the conclusions cannot be generalized. More SPs should be used as assessors and instructors in the educating medical students in the future.

## Conclusion

The present study demonstrated excellent scoring consistency between SPs and examiners, suggesting that using SPs directly as assessors was feasible after comprehensive and detailed pre-exam training and a well-designed rating scale. They can be used for teaching assessment and usual teaching and training, as well as to provide simulated and realistic clinical settings, thus creating favorable conditions for the comprehensive competence training and improvement of medical students.

## Data Availability

The datasets used and/or analyzed during the current study are available from the corresponding author upon reasonable request.
